# Shunt-related cerebrospinal fluid overdrainage – a multicentre consensus definition

**DOI:** 10.1007/s00701-026-06892-6

**Published:** 2026-05-07

**Authors:** S. Hornshøj Pedersen, A. Ammar, M. Czosnyka, Z. Czosnyka, C. Deopujari, F. Di Rocco, P. K. Eide, A. Grotenhuis, U. Kehler, H. Rekate, D. Rigamonti, L. Padayachy, G. Palandri, B. Pettorini, M. U. Schuhmann, E. Schmidt, T. S. Hansen, U. W. Thomale, A. K. Toma, M. Juhler

**Affiliations:** 1 Department of Neurosurgery, Copenhagen University Hospital, Copenhagen, Denmark; 2 Department of Neurosurgery, Faculty of Medicine, Delta University for Science and Technology, Gamasa, Egypt; 3 Department of Clinical Neurosciences, University of Cambridge, Cambridge, England; 4 Department of Neurosurgery, Bombay Hospital and Medical Research Centre, Bombay, India; 5 Department of Pediatric Neurosurgery, Woman-Mother-Child Hospital, Hospices Civils de Lyon, University of Lyon, Lyon, France; 6 Department of Neurosurgery, Oslo University Hospital-Rikshospitalet, Oslo, Norway; 7 Institute of Clinical Medicine, Faculty of Medicine, University of Oslo, Oslo, Norway; 8 K.G. Jebsen Centre for Brain Fluid Research, University of Oslo, Oslo, Norway; 9 Medical Centre, Radboud University, Nijmegen, The Netherlands; 10 Department of Neurosurgery, Asklepios Klinik Altona, Hamburg, Germany; 11 The Donald and Barbara Zucker Hofstra Northwell School of Medicine, Hempstead, NY USA; 12 Department of Neurosurgery, Johns Hopkins Medicine, Baltimore, USA; 13 Brain Tumour and Translational Neuroscience Centre, Department of Neurosurgery, University of Pretoria, Steve Biko Academic Hospital, Pretoria, South Africa; 14 Institute of Neurological Sciences of Bologna, IRCCS, Bellaria Hospital, Bologna, Italy; 15 Department of Neurosurgery, Alder Hey Children’s Hospital, Liverpool, England; 16 Section of Pediatric Neurosurgery, Department of Neurosurgery, University Hospital Tübingen, Tübingen, Germany; 17 Department of Neurosurgery, University Hospital of Toulouse, Toulouse, France; 18 Department of Neurosurgery, Aarhus University Hospital, Aarhus, Denmark; 19 Pediatric Neurosurgery, Charité Universitätsmedizin, Berlin, Germany; 20 Queen Square National Hospital for Neurology and Neurosurgery, London, England

**Keywords:** CSF overdrainage, Overdrainage definition, Consensus project, Hydrocephalus, Complication to VP shunting

## Abstract

**Background:**

Overdrainage of the cerebrospinal fluid (CSF) is a known complication of hydrocephalus shunt treatment. The concept of CSF overdrainage has evolved alongside advancements in shunt technology and hydrocephalus management. A systematic literature review identified 22 descriptions of CSF overdrainage, highlighting the lack of a universally accepted definition. To address this ambiguity, we conducted a multicentre consensus study to establish a clinically relevant definition of CSF overdrainage that can guide future research and clinical decision-making.

**Methods:**

A modified Delphi consensus process was conducted with 18 neurosurgeons and neuroscientists specialising in hydrocephalus from centres in Africa, Europe, India, and USA. Participants had at least ten years of clinical experience and/or significant publications in the field. The previous systematic literature review identified 32 clinical and radiological manifestations of CSF overdrainage, which were evaluated by the consensus-panel. The process included multiple rounds of structured questionnaires and virtual discussions. Consensus was achieved if a manifestation gained more than 70% support throughout the process. Finally, through iterative refinement the definitions were established.

**Results:**

The panel agreed that CSF overdrainage presents differently in various patient populations and should be defined by cardinal symptoms/findings rather than additional less frequent symptoms/findings. Three age-dependent definitions were established for paediatric patients: infants (age < 1 year), children with a growing skull (age 1 to 7 years), and children with a fixed skull (age > 7 years). A separate definition was formulated for adults (age ≥ 18 years). Across all age groups, CSF overdrainage was characterised by posture-related symptoms, primarily headache or in infants’ irritability consistent with headache relieved in supine position. Radiological findings include ventricular collapse (age < 18 years) and non-traumatic subdural collections (all age groups).

**Conclusion:**

Consensus-based definitions provide a standardised framework for future research and clinical management of hydrocephalus. It addresses the longstanding variability in diagnosis and treatment of CSF overdrainage in shunt-treated patients.

**Supplementary Information:**

The online version contains supplementary material available at 10.1007/s00701-026-06892-6.

## Introduction

Cerebrospinal fluid (CSF) shunting was first introduced in 1937 by Arne Thorkildsen as a groundbreaking treatment for hydrocephalus [16]. Early shunt designs were simple differential pressure valves without mechanisms to regulate CSF flow, and by the 1950s, clinicians began to observe a range of complications associated with excessive CSF drainage. These included subdural haematomas [3], slit ventricle syndrome (SVS) [8], and intracranial hypotension presenting with low-pressure headaches and cranial deformation [19].

The term ‘CSF overdrainage’ was formally introduced in 1978 [17] to describe this distinct clinical entity and has evolved alongside advances in the treatment of hydrocephalus and the understanding of shunt-related complications. Although later innovations such as adjustable differential pressure valves, variable hydrodynamic resistance valves and accessories, anti-siphon membrane devices and gravitational shunt assistants, have reduced the risk of CSF overdrainage, it remains a frequent complication to CSF shunt diverting procedures [13, 18, 40]. Nearly half a century after its initial description, the clinical and radiological manifestations of CSF overdrainage remain poorly defined. A recently conducted systematic literature review of CSF overdrainage in relation to ventriculo-peritoneal (VP) shunting, identified 22 different definitions of CSF overdrainage in the literature [29]. Based on these definitions, two descriptions of CSF overdrainage were suggested 1) CSF overdrainage in children is “*a persistent condition with clinical manifestations as postural dependent headache and vomiting, mood change/irritability, sunken fontanelle and decreasing head circumference, and/or radiological signs as slim ventricles/complete ventricular collapse, subdural hygroma/haematoma and overriding or fused cranial sutures*”, and 2) CSF overdrainage in adults is “*a persistent condition with clinical manifestations as postural dependent headache, nausea, and vomiting, and/or radiological signs as slim ventricles (with disproportionally wide cortical sulci) and subdural hygroma/haematoma*”. The review also showed that the published incidence of CSF overdrainage is extremely variable, which was interpreted as a consequence of a lacking universally agreed-upon definition [29].

In response to the lack of a standardised globally used definition, we conducted a multicentre consensus project, involving a panel of neurosurgeons/neuroscientists with a particular knowledge in the field of hydrocephalus. Our aim was to establish a clear and clinically relevant definition of CSF shunt-related overdrainage that can serve as a foundation for future clinical research and guide clinical decision-making in the management of hydrocephalus and potentially improve patient outcomes. Thus, this paper does not aim to address clinical strategy or decision making in case of CSF overdrainage.

## Method

The work was conducted using a modified Delphi process, a method designed to achieve consensus on a given subject through controlled feedback from a panel of participants representative of their profession [39]. The consensus process in this study comprised several steps: selection of participants, a predetermination of content and idea generation, rounds of individual anonymised questionnaires to capture participant’s opinions, and subsequent face-to-face discussions allowing clarification of disagreements. The level of anonymity was defined a priori to study start.

### Selection of participants

This work was initiated by the author group of the previously mentioned systematic review on CSF overdrainage [29]. The authors of the review each nominated 2 to 3 candidates to participate in the modified Delphi process. The number and geographic distribution of participants were determined in accordance with Delphi process recommendations [14, 22, 39] and time-zone logistics for online meetings. Inclusion criteria for participation were a background in neuroscience or neurosurgery with a minimum of ten years of clinical experience with hydrocephalus management and/or a substantial publication record within the field of hydrocephalus and CSF overdrainage. Participants were further selected to reflect a balanced distribution of clinical practice profiles: solely paediatric patients (*n* = 7), mixed paediatric and adult patients (*n* = 4), and solely adult patients (*n* = 7). The panel comprised 18 participants from Africa (*n* = 2), Europe (*n* = 13), India (*n* = 1), and USA (*n* = 2). The first author of this report was not included in the panel but moderated throughout the process.

### Predetermination of content and idea generation

The recently conducted systematic literature review [29] exploring definitions of CSF overdrainage in the literature was used as an idea generation to determine the content of the consensus process. In the review process, 32 different clinical and radiological manifestations were extracted, with non-specific headache and position dependent headache being the most frequently reported clinical symptoms and collapsed (slit) ventricles and subdural collections the most frequently reported radiological findings (Supplementary 1). The 32 manifestations of CSF overdrainage were used in the initial questionnaires (Q1) (Supplementary 2). Throughout the process, participants could suggest amendments to the extracted manifestations or new manifestations not included in the systematic literature review.

### Questionnaires and discussion meetings

The first encounter was an introduction meeting, whereat the participants shortly introduced themselves and their motivation for participating in the consensus process. Subsequently, the moderator presented the findings from the systematic literature review [29] and thus the predetermination of content, and the aim of the modified Delphi process. The discussion meetings were conducted in English and lasted 60 min each. The meetings were conducted online through Microsoft Teams with digital audio recording. At each meeting, the moderator introduced the agenda of the meeting and presented the anonymised results of the questionnaires answered prior to the meeting. Throughout the debate, the moderator used a prompting technique to ensure flow in the conversations to make the participants feel at ease, avoid unrelated tangential discussions, and group pressure for compromises. Following each session, an anonymised summary including clarification of disagreements and modified manifestations was distributed to all panel members. All participants were encouraged to contribute anonymised comments to the summary and on any included, excluded, or modified manifestations.

**Fig. 1:**
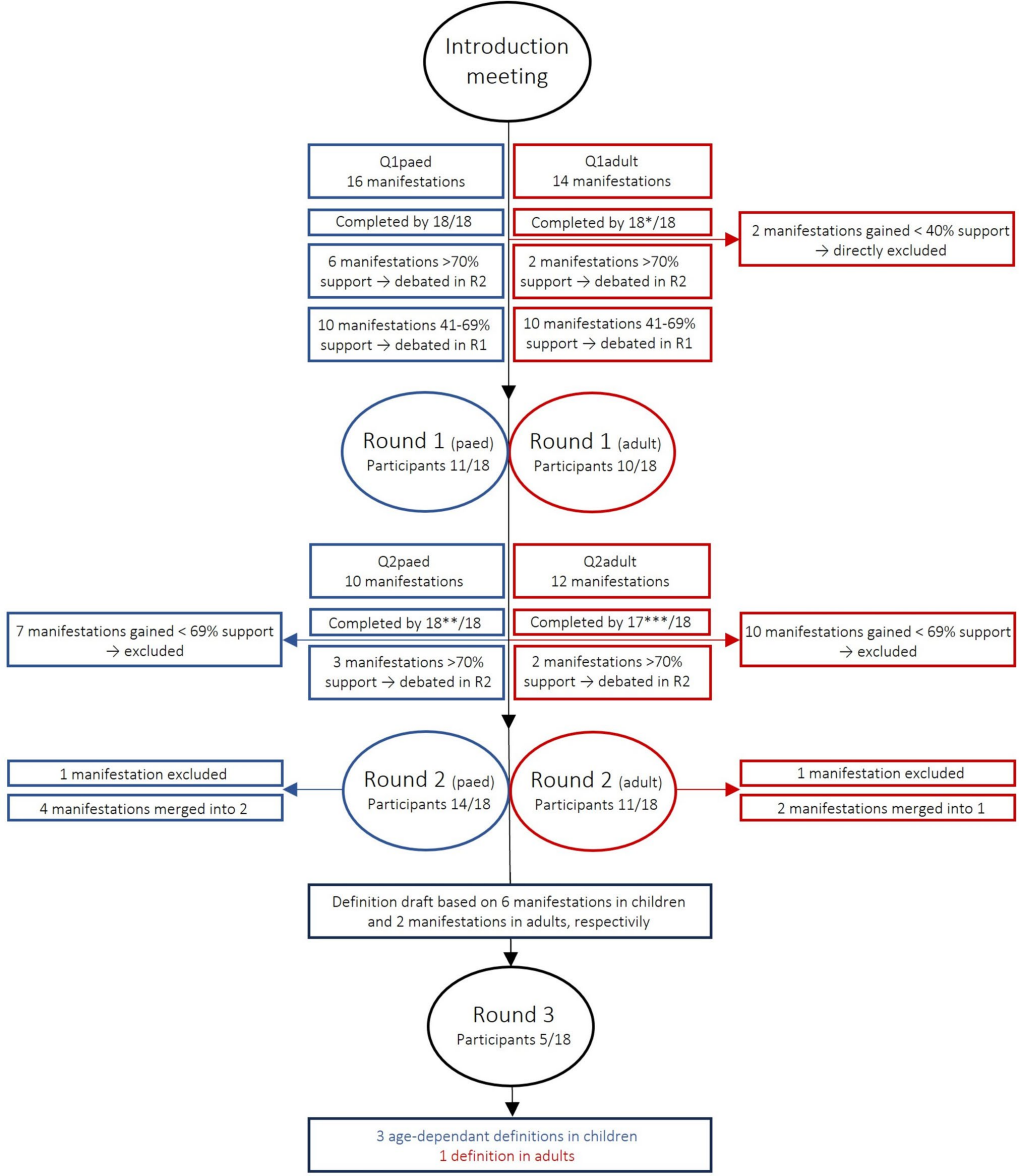
The modified Delphi process. The flowchart illustrates the modified Delphi process from the introduction meeting till round 3. Number of participants completing the questionaries and participating in each discussion meeting are listed. Blue; paediatric focus, red; adult focus. Following Q1, no paediatric- and two adult CSF overdrainage manifestations were directly excluded (wide cortical sulci, shunt patency studies), whereas six manifestations in children and two manifestations in adult were directly included to round 2 (*children*; position-dependent symptoms, subdural hygroma/haematoma, slim ventricles/decreased ventricle size/slit ventricles, complete ventricular collapse, overriding sutures, slit ventricle syndrome, and *adults*; position-dependent symptoms, subdural hygroma/haematoma). The 20 manifestations debated in round 1 can be seen in Table 1. Following Q2, seven paediatric- and ten adult manifestations were excluded (*children*; nausea, emesis/vomiting, fatigue, mood change, craniosynostosis, low ICP, incorrect shunt setting, and *adults*; nausea, emesis/vomiting, slim ventricles/decreased ventricle size/slit ventricles, decreased ventricle size, low ICP, risk of ventricular catheter obstruction due to collapse of the ventricular wall, new onset of unilateral ventricular collapse on site of the implanted shunt ± enlargement of the contralateral ventricle, improper shunt setting, an excessive siphoning effect, slit ventricle syndrome). The 13 manifestations in round 2 can be seen in Table 1. In round 2, one paediatric- and one adult manifestation were excluded (*children*; slit ventricle syndrome, and *adults*; low intracranial pressure syndrome). Further, six overlapping manifestations were merged into three. *One participant missed rating one of the paediatric manifestations. **One participant missed rating seven of the paediatric manifestations. ***One participant did not answer the questionnaire, while five participants missed rating each one manifestation. Q1-2paed: Questionnaire containing manifestations of CSF overdrainage in paediatric patients. Q1-2adult: Questionnaire containing manifestations of CSF overdrainage in adult patients. R1: round 1, R2: round 2, R3: round 3

The flowchart in Fig. 1 illustrates the different steps in the modified Delphi process and inclusion/exclusion of manifestations related to CSF overdrainage.

#### Round 1

The discussion meeting in round 1 was preceded by two questionnaires; ‘Q1paed’ with sixteen CSF overdrainage manifestations extracted from the systematic literature review defining CSF overdrainage in solely paediatric patients [29], and ‘Q1adult’ with fourteen CSF overdrainage manifestations extracted from the systematic literature review defining CSF overdrainage in solely adult patients [29], Supplementary 2. The participants were instructed to rate each manifestation on the Likert scale (strongly disagree (1 point)/disagree (2 points)/neither agree nor disagree (3 points)/agree (4 points)/strongly agree (5 points)), e.g. *“I find that headache is a clear manifestation of overdrainage in children, and thus should be included in the definition of overdrainage in paediatric patients”*. Hence, based on the ratings from the 18 participants a manifestation could achieve between 18 to 90 points. Manifestations with 69 to 90 points (more than 70% support) were included directly and debated in round 2 (*n* = 8), manifestations with 48 to 68 points were debated in round 1 (*n* = 20, Table 1), and manifestations with 18 to 47 points (less than 40% support) were directly excluded (*n* = 2), see Fig. 1.

**Table 1:** Debated manifestations of CSF overdrainage

**Discussion meeting, round 1**					
Children (all age groups)			Adults		
**Clinical symptoms**	**Clinical findings**	**Radiological findings**	**Clinical symptoms**	**Clinical findings**	**Radiological findings**
Headache	Craniosynostosis		Headache	Low ICP	Slim ventricles/slit ventricles/decreased ventricle size
Nausea	Decreasing head circumference		Nausea	Risk of shunt obstruction	
Emesis/vomiting	Sunken fontanelle		Emesis/vomiting	Improperly shunt settings	
Fatigue	Low ICP			Siphoning	
Mood change (e.g. irritability)	Incorrect shunt setting			Slit ventricle syndrome	
				Low intracranial pressure syndrome	
** Discussion meeting, round 2 **					
Children (all age groups)			Adults		
**Clinical symptoms**	**Clinical finding**	**Radiological findings**	**Clinical symptoms**	**Clinical findings**	**Radiological findings**
Posture-related headache	Overriding sutures	Subdural hygroma/haematoma	Headache	Low intracranial pressure syndrome	Subdural hygroma/haematoma
Posture-dependent symptoms	Decreasing head circumference	Slim ventricles/decreased ventricle size/slit ventricles	Posture-dependent symptoms		
	Sunken fontanelle	Complete ventricular collapse			
	Slit ventricle syndrome				
**Discussion meeting, round 3**					
Children (all age groups)			Adults		
**Clinical symptoms**	**Clinical findings**	**Radiological findings**	**Clinical symptoms**	**Clinical findings**	**Radiological findings**
Posture-related headache or following activity	Overriding sutures persistent after initial shunting	Subdural collections	Posture-related headache		Subdural collections
	A persistent and continuous decreasing head circumference with percentile down crossing	Slit ventricles or complete ventricular collapse without/nearly without visible CSF around the ventricular catheter			
	Sunken fontanelle persistent in supine position				

The table contains manifestations of CSF overdrainage subgrouped in clinical symptoms, clinical findings, and radiological findings debated at discussion meeting 1–3. Manifestations were modified throughout the process, e.g. from “Slim ventricles/decreased ventricle size/slit ventricles” to “Slit ventricles or complete ventricular collapse without/nearly without visible CSF around the ventricular catheter”

*Siphoning is a physical mechanism which describes the increased CSF flow in an upright position

*CSF* cerebrospinal fluid

*ICP* intracranial pressure

#### Round 2

Round 2 included two discussion meetings with focus on CSF overdrainage in paediatric patients and in adult patients, respectively. The meetings were preceded by the questionnaires ‘Q2paed’ with ten manifestations of CSF overdrainage in children, and ‘Q2adult’ with twelve manifestations of CSF overdrainage in adults (Supplementary 3). Manifestations were modified according to the discussion during the round 1 meeting. The participants were instructed to rate whether they disagreed (0 point) or agreed (1 point) that the given manifestation is representative for CSF overdrainage in paediatric/adult patients. Manifestations with less than 70% support were directly excluded (*n* = 17), while manifestations with more than 70% support were included and debated at the meeting (*n* = 5). In total, nine manifestations of CSF overdrainage in paediatric patients and four manifestations of CSF overdrainage in adult patients were debated in round 2, Table 1, Fig. 1.

#### Round 3

Based on the included and modified manifestations, definitions of CSF overdrainage in paediatric patients and in adult patients were constructed and forwarded to all participants. All participants were asked to review the definitions and provide feedback, either written or at the discussion meeting in round 3. The modified final definitions are presented in the result section.

## Results

The consensus-panel agreed that CSF overdrainage presents itself in numerous ways in different patients and that cardinal symptoms/findings should be distinguished from additional symptoms/findings. It was agreed that cardinal symptoms/findings are characterised as symptoms/findings present in the majority of shunt-treated patients with CSF overdrainage, whereas additional symptoms/findings are less frequent, but present in some patients with CSF overdrainage. The consensus-panel agreed to include only cardinal symptoms/findings in the definitions of CSF overdrainage.

Further, it became evident that CSF overdrainage in paediatric patients depends on the development of the skull and the child’s ability to walk, and thus the child’s primary daily postural position. This resulted in three age-dependent CSF overdrainage definitions for paediatric patients.

The main results of the modified Delphi-process are the four working definitions described below and listed in Table 2. Additional symptoms/findings debated throughout the consensus-process are found in Supplementary 1–3 and in Table 1.

**Table 2:** Consensus-based definitions of shunt-related CSF overdrainage

Age group	Clinical symptoms	Clinical findings			Radiological findings	
	Position-related irritability/headache	Sunken fontanel	Overriding sutures	Downward crossing of head circumference percentiles	Ventricular collapse	Subdural collections
< 1 yr	X	X	X	X	X	X
1 to 7 yr	X			X	X	X
> 7 to < 18 yr	X				X	X
> 18 yr	X					X

CSF overdrainage can be characterised by clinical symptoms/findings and/or by radiological findings. Symptoms/findings listed in the definitions are cardinal symptoms/findings, defined as being present in the majority of patients with CSF overdrainage. Not all listed in the definitions need to be present in a patient with CSF overdrainage. It applies for all age-groups that 1) patients experience a relief of clinical symptoms in supine position, and a worsening in upright position, and 2) the pathology of the subdural collection is non-traumatic, the collection can be either unilateral or bilateral, and present as a haematoma, a hygroma or a mix

### Definition of shunt-related CSF overdrainage in relation to a functional VP shunt in children with an open fontanel and soft sutures (less than 1 year of age)

CSF overdrainage in children less than 1 year of age is defined by clinical and/or radiological symptoms/findings, which may occur independently or in combination.

Clinical symptoms/findings include *position-related irritability* presumed to reflect headache, with a relief of symptoms in supine position and a worsening of symptoms in an upright position. This can be accompanied by a sunken fontanel (present in both supine and upright position), overriding sutures and a persistent deceleration of head growth with downward crossing percentiles.

Radiological findings include uni- or bilateral *ventricular collapse* (with absent or limited visible CSF around the ventricular catheter compared to previous imaging) and/or *subdural collections*. The subdural collections can present as haematoma, hygroma or a mix, are typically non-traumatic and may be uni- or bilateral.

CSF overdrainage in children less than 1 year of age can include other less common symptoms/findings in addition to the above listed.

### Definition of shunt-related CSF overdrainage in relation to a functional VP shunt in children with a continuous growing skull (1 to 7 years of age)

CSF overdrainage in children aged 1 to 7 years is defined by clinical and/or radiological symptoms/findings, which may occur independently or in combination.

Clinical symptoms/findings include *position-related headache* (and/or obvious discomfort for children at age 1 to 2 years old/without a verbal function) with a relief of symptoms in supine position, and a worsening of symptoms in an upright position. This can be accompanied by a persistent deceleration of head growth with downward crossing percentiles.

Radiological findings include uni- or bilateral *ventricular collapse* (with absent or limited visible CSF around the ventricular catheter compared to previous imaging) and/or *subdural collections*. The subdural collections can present as haematoma, hygroma or a mix, are typically non-traumatic and may be uni- or bilateral.

CSF overdrainage in children aged 1 to 7 can include other less common symptoms/findings in addition to the above listed.

### Definition of shunt-related CSF overdrainage in relation to a functional VP shunt in children with a fixed-sized skull (older than 7 years of age)

CSF overdrainage in children older than 7 years of age is defined by clinical and/or radiological symptoms/findings, which may occur independently or in combination.

Clinical symptoms include *position-related headache* with a relief of symptoms in supine position, and a worsening of symptoms in an upright position.

Radiological findings include uni- or bilateral *ventricular collapse* (with absent or with limited visible CSF around the ventricular catheter compared to previous imaging) and/or *subdural collections*. The subdural collections can present as haematoma, hygroma or a mix, are typically non-traumatic and may be uni- or bilateral.

CSF overdrainage in children older than 7 years can include other less common symptoms/findings in addition to the above listed.

### Definition of shunt-related CSF overdrainage in relation to a functional VP shunt in adults (18 years or above)

CSF overdrainage in adults is defined by clinical and/or radiological symptoms/findings, which may occur independently or in combination.

Clinical symptoms include *position-related headache* with a relief of symptoms in supine position, and a worsening of symptoms in an upright position.

Radiological findings include *subdural collections*. The subdural collections can present as haematoma, hygroma or a mix, are typically non-traumatic and may be uni- or bilateral.

CSF overdrainage in adults can include other less common symptoms/findings in addition to the above listed.

## Discussion

The term ‘CSF overdrainage’ has been used to describe symptoms related to excessive CSF drainage in relation to shunt diverting procedures for nearly half a century. Although the phenomenon is well-known, no universally agreed-upon definition exits. Symptomatic overdrainage is estimated to affect 10 to 20% of long-term shunted adults [13], though rates in the literature vary widely [13, 40].

Through a modified Delphi process, including an international panel consisting of 18 participants with recognised knowledge within the field of hydrocephalus, we suggest four consensus-based age-related definitions of CSF overdrainage in patients with a VP shunt. We found an inverse relationship between age of the patient and complexity of the definition. Hence, the panel agreed that the definition of CSF overdrainage in infants should include six cardinal symptoms/findings, whereas only two cardinal symptoms/findings were included in the definition of CSF overdrainage in adults. The panel is aware that cardinal symptoms/findings are present in most, but not all patients with CSF overdrainage, and that additional symptoms/findings of CSF overdrainage can be seen in some patients with or without cardinal symptoms/findings.

## Manifestations of CSF overdrainage

### Children

In paediatric patients, CSF overdrainage primarily presents as a chronic condition. Symptoms are most pronounced in the upright position, reflecting gravitational decrease in intracranial pressure (ICP), but may also be triggered or worsened by physical activity. The youngest children and children without a verbal function often show posture-related irritability or discomfort, likely due to headache, while older children primarily complain of headache as *cardinal clinical symptom*.

In infancy, a low upright ICP during cranial growth can lead to a deflated fontanelle, overriding sutures, and a relatively diminished increase in head circumference, resulting in downward crossing of growth percentiles [6, 25]. In children with a closed fontanelle but ongoing cranial growth, head circumference may remain small for age due to a reduced cranial growth, again reflected by downward percentile trends, even in the absence of overt ventricular changes. Early recognition of CSF overdrainage thus relies on monitoring these *cardinal clinical findings* [20].

Across all paediatric age groups, the *cardinal radiological findings* are subdural collections and/or ventricular collapse. Though the ventricular size in some cases remains unchanged likely due to impaired flexibility of the brain tissue, most cases will present a progressive decrease in ventricular width developing towards a slit-like configuration as an indicator of CSF overdrainage.

As many children can partly compensate for gravitational ICP differences, overt clinical signs may be subtle. In such cases, diagnosis relies on *additional clinical symptoms*, such as nausea, emesis, fatigue, or mood changes associated with changes in posture or during/following severe physical activity.

Persistent CSF overdrainage during infancy may lead to *additional clinical findings*, including microcephaly, abnormal cranial shape due to restricted skull growth, calvarial thickening, and secondary Chiari malformation related to reduced intracranial volume [20]. These changes typically develop gradually if left uncorrected, and chronic overdrainage may progress to SVS [20]. Owing to the diverse aetiologies requiring shunt treatment in children, brain growth potential, growth rate, and venous pressure vary considerably. These factors influence head circumference and ventricular size and must be considered alongside shunt-related effects.

### Adults

Despite different aetiologies across adult age groups (e.g. in shunted patients aged 40 vs 80 years), the consensus-panel agreed that the cardinal symptoms/findings in adults with CSF overdrainage can be captured within a single definition. Consistent with CSF overdrainage in older children, the *cardinal clinical symptom* of CSF overdrainage in adults is posture-related headache. The *cardinal radiological finding* is non-traumatic subdural fluid collections, often without significant ventricular collapse. As adults have fixed cranial vaults and lower cerebrospinal compliance, they are more susceptible to the development of subdural collections and traction on the bridging veins, which may contribute further to the collections. Notably many adult patients with radiological signs of CSF overdrainage remain asymptomatic.

For all adult age groups, posture-related headache may be accompanied by *additional clinical symptoms* such as posture-related nausea, emesis or dizziness. Adults who were shunted early in life and develop long-term CSF overdrainage may present with *additional clinical- or radiological findings* typically seen in paediatric patients, including craniocerebral disproportion, Chiari malformations, or SVS. In contrast, adults with secondary hydrocephalus usually present with the above-mentioned *additional clinical symptoms*, but rarely with radiological changes. Older adults with normal pressure hydrocephalus (NPH) may develop above-mentioned *additional clinical symptoms* but are especially prone to subdural collections, due to larger ventricles, reduced compliance, and more fragile bridging veins, particularly when managed with low-pressure valve settings or without antisiphon devices.

### Syndromes

Definitions of CSF overdrainage in the systematic literature review [29] identified SVS and low intracranial pressure syndrome as manifestations of overdrainage. However, description of both conditions varied, and no consensus-based definition exists [21, 28, 33, 36].

Children with a longstanding history of shunt treatment often present with ventricular collapse and are thus in risk of intermittent obstruction of the proximal catheter due to catheter impingement from a continuous inverse flow into the catheter. Catheter occlusion may lead to an increased pressure gradient both in intraventricular- and external CSF spaces, which is believed to cause occlusion of the bridging veins at the convexity, leading to intracranial venous congestion and thereby an increase of blood volume in the brain. These pathophysiological sequences prevent enlargement of the ventricular system. The condition presents clinically with slow refill of the shunt pumping device and intermittent severe headache, nausea, vomiting or even reduced consciousness. Within variations, this combination of clinical symptoms, clinical findings and radiological findings is called SVS [1, 7, 28, 35]. The consensus-panel agreed that since 1) CSF overdrainage is one of the pathophysiologic mechanisms of SVS and 2) the literature does not agree on the description, SVS itself cannot be included as a cardinal finding of CSF overdrainage.

A few studies in the literature have termed posture-dependent worsening of a variety of clinical symptoms related to CSF overdrainage as low intracranial pressure syndrome [21, 33, 36]. As none of the participants in the panel were familiar with the term “low intracranial pressure syndrome” caused by shunting, the consensus-panel agreed that it was considered too rare to be included as a cardinal finding of CSF overdrainage.

## Valve types and CSF overdrainage

The occurrence of CSF overdrainage is associated with the type of valve used in the CSF diversion treatment [10]. Shunts with some sort of built-in or added overdrainage protection are generally preferable from the initial shunt implantation to avoid or reduce the risk of subsequent CSF overdrainage. Technologies intended to prevent, reverse or ameliorate CSF overdrainage include adjustable differential pressure valves, anti-siphon components and flow-regulated valves. More sophisticated technology includes gravitational valves with variable opening pressure depending on body position and adjustable flow regulated valves.

Individual differences in CSF pressure dynamics may influence the risk of CSF overdrainage and the literature does not support advocating any specific valve type to avoid it [2, 10, 24, 38]. Moreover, the literature does not allow conclusions regarding whether the risk of CSF overdrainage differs according to shunt placements (ventriculo-atrial, ventriculo-peritoneal, lumbo-peritoneal or ventriculo-sagittal sinus) [23].

## ICP measurement, regulation, and physiology of overdrainage

Four of the 22 studies included in the systematic literature review used as an idea generator [29], stated ‘low ICP’ as an indicator of CSF overdrainage. The consensus-panel agreed that even though low ICP values, particularly in upright position, can be useful to support the suspicion of CSF overdrainage, results of an invasive measurement method which is not globally available, cannot be a cardinal finding and thus included in the consensus-definitions. However, measurement or monitoring of ICP can be extremely useful in confirming a clinical suspicion of CSF overdrainage.

### ICP measurement

In adults, normal ICP in supine position ranges from 0.9 to 16.3 mmHg [27], while values in children seem to be slightly higher [11, 30]. In non-shunted adults/children, a postural change from horizontal to vertical will immediately induce a physiological decrease in ICP, often with stabilised values slightly lower than 0 mm Hg [4, 5, 26].

In shunted patients, slightly negative ICP in supine position is indicative of CSF overdrainage, and upright values lower than −10 mmHg are strongly indicative of CSF overdrainage [31, 37, 41]. Evaluating postural changes in ICP, e.g. by tilt-test or sit-up test, is particularly informative in cases of CSF overdrainage. The pressure drop will often be steeper and followed by gradual slow decrease in ICP which stabilises at values below −10 mmHg [12].

Invasive monitoring remains the most reliable method for assessing ICP. In shunted patients, ICP can be measured through a shunt pre-chamber or burr-hole reservoir using a fine-gauge (25 G recommended) needle, via a pressure sensor connected to the shunt system or by a separately implanted sensor. In all cases, waveform pulsatility is essential (“no pulse, no pressure” principle [15]). Non-invasive approaches, such as optic nerve sheath diameter ultrasound and transcranial Doppler-based estimation, may supplement invasive methods with consecutive measurements with the patient as his/her own control are needed [34].

### ICP regulation

Normal ICP regulation depends on the balance between brain, CSF, and cerebral blood volumes. These relationships follow a sigmoidal pressure–volume (P–V) curve, with compensatory reserve quantified by the RAP index (correlation coefficient between slow fluctuations (20 s to 2 min period) of mean ICP and amplitude of pulsatile ICP waveform) [32]. In healthy physiology, small volume changes are buffered by CSF redistribution and venous blood displacement.

Shunt implantation alters this regulation by providing a low-resistance outflow pathway. This shifts the entire P–V curve downward, often reducing pulse amplitude and lowering measured resistance to CSF outflow. Compensatory reserve increases, reflecting increased ability to buffer volume fluctuations with markedly decreasing RAP index. In patients with CSF overdrainage, ICP regulation becomes dominated by gravitational forces and unidirectional flow characteristics of the shunt valve. These factors prevent normal volume-pressure recovery, leading to sustained negative pressures and increased intracranial compliance over time.

### Causes and physiology of overdrainage

CSF overdrainage following shunting is a consequence of excessive CSF drainage and occurs through different mechanisms: 1) passive posture-related siphoning, 2) activity-related increase of differential pressure between the intracranial space and the peritoneal cavity, 3) slow, high-amplitude vasogenic intracranial pressure oscillations, and less common, 4) pump-mediated overdrainage due to repeated manual compression of a prechamber [9]. Posture-related siphoning is the most common mechanism. In the upright position, a hydrostatic column adds ~ 10 mmHg or more to the effective pressure gradient across the shunt. Given the low resistance of most shunts (3 to 6 mmHg min/ml), upright flow rates may reach 1.5 to 3 ml/min, far exceeding normal CSF production. Slow vasogenic waves (> 5 to 7 mmHg) further promote unidirectional CSF loss, as CSF expelled during wave peaks cannot return at troughs. Chronic exposure to these dynamics can shift compliance characteristics and perpetuate negative ICP.

## Limitations

Several limitations of this modified Delphi process should be acknowledged. First, as with any consensus-based method involving face-to-face meetings, there is an inherent risk of group thinking, where the discussion dynamic may influence individual responses. This was mitigated by the moderator actively facilitating balanced discussion and by distributing an anonymised summary following each meeting and an anonymised new questionnaire prior to the next meeting, in which all proposed modifications were presented without attribution, allowing participants to reconsider their positions independently.

Second, there is a risk that participants with expertise outside the age group under discussion may be more susceptible to being persuaded by those with direct expertise in that group. The moderator sought to address this by ensuring equal opportunity for contribution across all participants, regardless of their primary practice profile.

Third, the consensus-panel reached agreement that a single definition could adequately capture cardinal symptoms/findings across adult aetiologies ranging from secondary hydrocephalus to NPH. However, the overall profile, including additional symptoms/findings differs between aetiologies. This clinically relevant distinction should be considered when applying the definition in practice, and the absence of aetiology-specific definitions for adults represents a limitation of the current framework.

Finally, it was outside the scope of this work to address clinical decisions dealing with CSF overdrainage. This important question needs to be separately examined by a new consensus process and clinical studies specifically aimed at this issue. This includes addressing the controversy whether to treat patients with radiological CSF overdrainage without or with discrete symptoms.

## Conclusion

This consensus-based Delphi study establishes four age-specific and clinically applicable definitions of shunt-related CSF overdrainage. The findings demonstrate an inverse relationship between age of the patient and complexity of the definition of CSF overdrainage. The consensus-panel agreed that only cardinal symptoms/findings should form the basis of a formal definition, while additional symptoms/findings may support clinical judgement. The consensus definitions provide an essential framework for future research, improve diagnostic precision, and may contribute to more standardised clinical management and reporting in patients with suspected CSF overdrainage.

It must be emphasised, however, that these definitions are founded upon consensus and not on scientific evidence. As such, they represent a necessary but preliminary step toward standardisation, and their clinical validity has yet to be established. Formal validation will however be challenging, as it will require long-term, multicentre data collection, and the identification of appropriate outcome parameters across different age groups remains inherently problematic. As clinical experience with these definitions accumulates, a follow-up consensus process would be a valuable means of refining and validating the current framework in light of emerging evidence.

## Supplementary Information

Below is the link to the electronic supplementary material.

ESM 1 Supplementary Material 1 (PDF 1.49 MB)

## Data Availability

Questionnaire data and interview recordings are available from the corresponding author on reasonable request.
