# Supplementary material for: Shunt-related cerebrospinal fluid overdrainage – a multicentre consensus definition
Source: Acta Neurochir (Wien). 2026 May 7;168(1):147. doi: 10.1007/s00701-026-06892-6 (PMC13315515; doi:10.1007/s00701-026-06892-6)
Supplement: Supplementary file 1 — Supplementary Material 1 (PDF 1.49 MB) [file 701_2026_6892_MOESM1_ESM.pdf]

Supplementary material

Supplementary 1

The table contains all extracted manifestations from the systematic literature review[1] and how frequently they were presented.

Population / included papers	Clinical symptoms													
	Headache	Position-dependent symptoms	Nausea	Emesis/vomiting	Fatique	Mood change	Altered consioness	Decreased consentration	Incontinens	Loss of appetite	Body pain	Restlessness	Neurological symptoms	Vertigo/dizziness
Pediatric (7 papers)	3	3	1	2	1	2	-	-	-	-	-	-	-	-
Mixed (2 papers)	2	2	2	1	1	-	1	1	1	1	1	1	-	1
Adults (12 papers)	8	4	6	5	-	-	-	-	-	-	-	-	-	-
Unknown (1 paper)	1	-	-	1	-	-	1	-	-	-	-	-	1	-
Frequency of symptoms in total [%]	66.7	42.9	42.9	42.9	9.5	9.5	9.5	4.8	4.8	4.8	4.8	4.8	4.8	4.8

Population / included papers	Radiological findings			Clinical findings			
	Subdural hygroma/hematoma	Slit ventricles / complete ventricular collaps	Wide cortical sulci	Craniosynostosis	Decreasing headcircumference	Overriding sutures	Sunken fontanelle
Pediatric (7 papers)	3	4	-	1	1	1	1
Mixed (2 papers)	1	2	1	-	-	-	-
Adults (12 papers)	10	7	1	-	-	-	-
Unknown (1 paper)	-	-	-	-	-	-	-
Frequency of symptoms in total [%]	66.7	61.9	9.5	4.8	4.8	4.8	4.8

Supplementary 2

Q1paed contains manifestations of CSF overdrainage extracted from the systematic literature review defining CSF overdrainage in solely paediatric patients. The participants were asked to rate six clinical manifestations, seven radiological manifestations, one measurable manifestation, one manifestation related to shunt complication and one syndrome.

Q1adult contains manifestations of CSF overdrainage extracted from the systematic literature review defining CSF overdrainage in solely adult patients. The participants were asked to rate four clinical manifestations, three radiological manifestations, two measurable manifestations, three manifestations related to shunt complication and two syndromes.

Each questionnaire was initiated with a brief instruction asking the participant to rate each manifestation on the Likert scale, based on whether they find that the given manifestation was representative of CSF overdrainage and thus should be included in the final consensus definition. It was highlighted that the participant should choose one answer per manifestation.

Pediatric patients

Instructions

The manifestations listed below are extracted from those 6 papers defining OD in solely pediatric patients. The manifestations are thus specific for pediatric patients only.

You are asked to list whether you agree or disagree (on the Likert scale) that the given manifestation is representative of OD in pediatric patients and thus should be included in the final consensus definition.

Please choose one answer to all manifestations.

At the first discussion meeting we will discuss those manifestations the majority agrees or strongly agrees with.

	Choose one answer	
Clinical manifestations		
I find that ”**headache**” is a clear manifestation of overdrainage in children, and thus should be included in the definition of overdrainage in pediatric patients.	Strongly disagree	
	Disagree	
	Neither agree or disagree	
	Agree	
	Strongly agree	
I find that ”**position-dependent symptoms**” is a clear manifestation of overdrainage in children, and thus should be included in the definition of overdrainage in pediatric patients.	Strongly disagree	
	Disagree	
	Neither agree or disagree	
	Agree	
	Strongly agree	
I find that ”**nausea**” is a clear manifestation of overdrainage in children, and thus should be included in the definition of overdrainage in pediatric patients.	Strongly disagree	
	Disagree	
	Neither agree or disagree	
	Agree	
	Strongly agree	
I find that ”**emesis/vomiting**” is a clear manifestation of overdrainage in children, and thus should be included in the definition of overdrainage in pediatric patients.	Strongly disagree	
	Disagree	
	Neither agree or disagree	
	Agree	
	Strongly agree	
I find that ”**fatigue**” is a clear manifestation of overdrainage in children, and thus should be included in the definition of overdrainage in pedatric patients.	Strongly disagree	
	Disagree	
	Neither agree or disagree	
	Agree	
	Strongly agree	
I find that ”**mood change (e.g. irritability)**” is a clear manifestation of overdrainage in children, and thus should be included in the definition of overdrainage in pediatric patients.	Strongly disagree	
	Disagree	
	Neither agree or disagree	
	Agree	
	Strongly agree	
Radiological manifestations		
I find that ”**subdural hygroma/hematoma**” is a clear manifestation of overdrainage in children, and thus should be included in the definition of overdrainage in pediatric patients.	Strongly disagree	
	Disagree	
	Neither agree or disagree	
	Agree	
	Strongly agree	
I find that ”**slim ventricles/decreased ventricle size/slit ventricles**” is a clear manifestation of overdrainage in children, and thus should be included in the definition of overdrainage in pediatric patients.	Strongly disagree	
	Disagree	
	Neither agree or disagree	
	Agree	
	Strongly agree	
I find that ”**craniosynostosis**” is a clear manifestation of overdrainage in children, and thus should be included in the definition of overdrainage in pediatric patients.	Strongly disagree	
	Disagree	
	Neither agree or disagree	
	Agree	
	Strongly agree	
	Strongly disagree	
	Disagree	

Pediatric patients

I find that "**complete ventricular collapse**" is a clear manifestation of overdrainage in children, and thus should be included in the definition of overdrainage in pediatric patients.	Neither agree or disagree	
	Agree	
	Strongly agree	
I find that "**decreasing head circumference**" is a clear manifestation of overdrainage in children, and thus should be included in the definition of overdrainage in pediatric patients.	Strongly disagree	
	Disagree	
	Neither agree or disagree	
	Agree	
	Strongly agree	
I find that "**overriding sutures**" is a clear manifestation of overdrainage in children, and thus should be included in the definition of overdrainage in pediatric patients.	Strongly disagree	
	Disagree	
	Neither agree or disagree	
	Agree	
	Strongly agree	
I find that "**sunken fontanelle**" is a clear manifestation of overdrainage in children, and thus should be included in the definition of overdrainage in pediatric patients.	Strongly disagree	
	Disagree	
	Neither agree or disagree	
	Agree	
	Strongly agree	
Measurable manifestation		
I find that "**low ICP**" is a clear manifestation of overdrainage in children, and thus should be included in the definition of overdrainage in pediatric patients.	Strongly disagree	
	Disagree	
	Neither agree or disagree	
	Agree	
	Strongly agree	
Manifestations related to shunt complication		
I find that "**incorrect shunt settings**" is a clear manifestation of overdrainage in children, and thus should be included in the definition of overdrainage in pediatric patients.	Strongly disagree	
	Disagree	
	Neither agree or disagree	
	Agree	
	Strongly agree	
Syndrome		
I find that "**slit ventricles syndrome**" is a clear manifestation of overdrainage in children, and thus should be included in the definition of overdrainage in pediatric patients.	Strongly disagree	
	Disagree	
	Neither agree or disagree	
	Agree	
	Strongly agree	

Adult patients

Instructions

The manifestations listed below are extracted from those 12 papers defining OD in solely adult patients. The manifestations are thus specific for adult patients only.

You are asked to list whether you agree or disagree (on the Likert scale) that the given manifestation is representative of OD in adult patients and thus should be included in the final consensus definition.

Please choose one answer to all manifestations.

At the first discussion meeting we will discuss those manifestations the majority agrees or strongly agrees with.

	Choose one answer	
Clinical manifestations		
I find that ”**headache**” is a clear manifestation of overdrainage in adults, and thus should be included in the definition of overdrainage in adult patients.	Strongly disagree	
	Disagree	
	Neither agree or disagree	
	Agree	
	Strongly agree	
I find that ”**position-dependent symptoms**” is a clear manifestation of overdrainage in adults, and thus should be included in the definition of overdrainage in adult patients.	Strongly disagree	
	Disagree	
	Neither agree or disagree	
	Agree	
	Strongly agree	
I find that ”**nausea**” is a clear manifestation of overdrainage in adults, and thus should be included in the definition of overdrainage in adult patients.	Strongly disagree	
	Disagree	
	Neither agree or disagree	
	Agree	
	Strongly agree	
I find that ”**emesis/vomiting**” is a clear manifestation of overdrainage in adults, and thus should be included in the definition of overdrainage in adult patients.	Strongly disagree	
	Disagree	
	Neither agree or disagree	
	Agree	
	Strongly agree	
Radiological manifestations		
I find that ”**subdural hygroma/hematoma**” is a clear manifestation of overdrainage in adults, and thus should be included in the definition of overdrainage in adult patients.	Strongly disagree	
	Disagree	
	Neither agree or disagree	
	Agree	
	Strongly agree	
I find that ”**slim ventricles/decreased ventricle size/slit ventricles**” is a clear manifestation of overdrainage in adults, and thus should be included in the definition of overdrainage in adult patients.	Strongly disagree	
	Disagree	
	Neither agree or disagree	
	Agree	
	Strongly agree	
I find that ”**wide cortical sulci**” is a clear manifestation of overdrainage in adults, and thus should be included in the definition of overdrainage in adult patients.	Strongly disagree	
	Disagree	
	Neither agree or disagree	
	Agree	
	Strongly agree	
Measurable manifestations		
I find that ”**low ICP**” is a clear manifestation of overdrainage in adults, and thus should be included in the definition of overdrainage in adult patients.	Strongly disagree	
	Disagree	
	Neither agree or disagree	
	Agree	
	Strongly agree	
I find that ”**shunt patency studies**” is a clear manifestation of overdrainage in adults, and thus should be included in the definition of overdrainage in adult patients.	Strongly disagree	
	Disagree	
	Neither agree or disagree	
	Agree	
	Strongly agree	
Manifestations related to shunt complications		
	Strongly disagree	
	Disagree	

Adult patients

I find that ”**risk of shunt obstruction**” is a clear manifestation of overdrainage in adults, and thus should be included in the definition of overdrainage in adult patients.	Neither agree or disagree	
	Agree	
	Strongly agree	
I find that ”**improperly shunt settings**” is a clear manifestation of overdrainage in adults, and thus should be included in the definition of overdrainage in adult patients.	Strongly disagree	
	Disagree	
	Neither agree or disagree	
	Agree	
	Strongly agree	
I find that ”**siphoning**” is a clear manifestation of overdrainage in adults, and thus should be included in the definition of overdrainage in adult patients.	Strongly disagree	
	Disagree	
	Neither agree or disagree	
	Agree	
	Strongly agree	
Syndromes		
I find that ”**slit ventricles syndrome**” is a clear manifestation of overdrainage in adults, and thus should be included in the definition of overdrainage in adult patients.	Strongly disagree	
	Disagree	
	Neither agree or disagree	
	Agree	
	Strongly agree	
I find that ”**low intracranial pressure syndrome**” is a clear manifestation of overdrainage in adults, and thus should be included in the definition of overdrainage in adult patients.	Strongly disagree	
	Disagree	
	Neither agree or disagree	
	Agree	
	Strongly agree	

Supplementary 3

Q2paed contains the ten manifestations of CSF overdrainage modified from round 1. The participants were asked to rate seven clinical manifestations, one radiological manifestation, one measurable manifestation and one manifestation related to shunt complication. Each manifestation was followed by a short explanation highlighting the debate from the discussion meeting, round 1.

Q2adult contains the twelve manifestations of CSF overdrainage modified from round 1. The participants were asked to rate three clinical manifestations, two radiological manifestations, one measurable manifestation, four manifestations related to shunt complication and two syndromes. Like in Q2paediatric, each manifestation was followed by a short explanation highlighting the debate from the discussion meeting, round 1.

Each questionnaire was initiated with a brief instruction asking the participant to rate whether they agreed or disagreed that the given manifestation was representative of CSF overdrainage and thus should be included in the final consensus definition. Further, the participant was asked to consider whether CSF overdrainage could be present without the given manifestation, or if the manifestation was in fact a cardinal symptom/finding.

Paediatric patients

Instructions
The 10 manifestations listed below are those **paediatric** manifestations rated as 'yellow' and discussed at the first discussion meeting.<br><br>After each manifestation, the main discussion-points from the meeting are *described*.<br><br>I will ask you to list <u>whether you agree or disagree that the given symptom/manifestation is representative of OD in paediatric patients</u> and thus should be considered for inclusion in the final consensus definition.<br><br>Please choose one answer to all manifestations. I will 'translate' *disagree* to the red group and *agree* to the green group. All symptoms/manifestations in the green group (from this rating and from the previous ratings) will be discussed at the next meetings and a symptom/manifestation is therefore not guaranteed inclusion in the final definition, but rather guaranteed inclusion in the final discussion of which symptoms/manifestations should be included in the consensus definition.<br><br>I will ask you to consider the following - <u>if OD can be present without</u> the given symptom/manifestation, shall the symptom/manifestation then be included in the consensus definition?<br><br>I will suggest that the final definition is <u>representative for most paediatric patients with OD</u> – meaning, that if a symptom/manifestation is not present in most patients with OD, the symptom/manifestation should not be included in the consensus definition but discussed in the discussion section in the paper. However, feel free to disagree.<br><br>**Deadline: 26th of April**

	Choose one answer	
Clinical manifestations		
I find that "**posture-related headache**" is a clear manifestation of overdrainage in children, and thus should be included in the definition of overdrainage in paediatric patients.<br><br>*The headache worsens in the upright position and/or following activity.*<br>*Often the patient experiences a relieve of the headache in supine position.*	Disagree<br><br>Agree	
I find that "**nausea**" is a clear manifestation of overdrainage in children, and thus should be included in the definition of overdrainage in paediatric patients.<br><br>*The nausea worsens in the upright position and/or following activity. It can be followed by vomiting.*	Disagree<br><br>Agree	
I find that "**emesis/vomiting**" is a clear manifestation of overdrainage in children, and thus should be included in the definition of overdrainage in paediatric patients.<br><br>*The vomiting worsens in the upright position and/or following activity.*	Disagree<br><br>Agree	
I find that "**fatique**" is a clear manifestation of overdrainage in children, and thus should be included in the definition of overdrainage in paediatric patients.<br><br>*The fatique worsens after activity or is directly correlated to activity. Improves when the child is laying down.*	Disagree<br><br>Agree	
I find that "**mood change**" is a clear manifestation of overdrainage in children, and thus should be included in the definition of overdrainage in paediatric patients.<br><br>*Following activity, and thus, might, correlated to above mentioned activity-dependant-symptoms.*	Disagree<br><br>Agree	
I find that "**decreasing head circumference**" is a clear manifestation of overdrainage in children, and thus should be included in the definition of overdrainage in paediatric patients.<br><br>*A persistent and continuous decreasing head circumference with percentile down crossing.*	Disagree<br><br>Agree	
I find that "**sunken fontanelle**" is a clear manifestation of overdrainage in children, and thus should be included in the definition of overdrainage in paediatric patients.<br><br>*A pronounced sunken fontanelle in upright as well as in supine position, present even after the initial shunting.*	Disagree<br><br>Agree	
Radiological manifestation		
I find that "**craniosynostosis**" is a clear manifestation of overdrainage in children, and thus should be included in the definition of overdrainage in paediatric patients.<br><br>*The craniosynostosis should be 'new onset' and thus not present at birth.*	Disagree<br><br>Agree	
Measurable manifestation		

Paediatric patients

I find that ”**low ICP**” is a clear manifestation of overdrainage in children, and thus should be included in the definition of overdrainage in paediatric patients.<br><br>*What is normal age-related ICP?*<br>*Shall an invasive measurement be included in the definition, or optional for the physician?*<br>*Is low ICP in fact just a sign that the patient has underwent ICP measurement, and thus not a significant issue in terms of an OD-definition?*<br>*One could argue that the ICP-drop seen in a ‘situp test’ is more accurate that than the absolute ICP value.*	Disagree	
	Agree	
Manifestation related to shunt complication		
I find that ”**incorrect shunt settings**” is a clear manifestation of overdrainage in children, and thus should be included in the definition of overdrainage in paediatric patients.<br><br>*One could argue that incorrect shunt setting might be a reason to OD, and not a manifestation of OD.*<br>*What is correct a correct shunt setting?*<br>*The correct shunt setting changes with age and size of the child. We could therefor suggest different settings to different scenarios in the discussion?*	Disagree	
	Agree	

Adult patients

Instructions

The 12 manifestations listed below are those **adult** manifestations rated as 'yellow' and discussed at the discussion meeting.

After each manifestation, the main discussion-points from the meeting are *described*.

I will ask you to list whether you agree or disagree that the given symptom/manifestation is representative of OD in adult patients and thus should be considered for inclusion in the final consensus definition.

Please choose one answer to all manifestations. I will 'translate' *disagree* to the red group and *agree* to the green group. All symptoms/manifestations in the green group (from this rating and from the previous ratings) will be discussed at the next meetings and a symptom/manifestation is therefore not guaranteed inclusion in the final definition, but rather guaranteed inclusion in the final discussion of which symptoms/manifestations should be included in the consensus definition.

I will ask you to consider the following - if OD can be present without the given symptom/manifestation, shall the symptom/manifestation then be included in the consensus definition?

I will suggest that the final definition is representative for most adult patients with OD – meaning, that if a symptom/manifestation is not present in most patients with OD, the symptom/manifestation should not be included in the consensus definition but discussed in the discussion section in the paper. However, feel free to disagree.

Deadline: 26th of April

	Choose one answer	
Clinical manifestations		
I find that "**headache**" is a clear manifestation of overdrainage in adults, and thus should be included in the definition of overdrainage in adult patients.<br><br>*The headache is often ortostatic, and thus with a relieve in supine position.*	Disagree	
	Agree	
I find that "**nausea**" is a clear manifestation of overdrainage in adults, and thus should be included in the definition of overdrainage in adult patients.<br><br>*The nausea worsens in the upright position and/or following activity. It can be followed by vomiting.*	Disagree	
	Agree	
I find that "**emesis/vomiting**" is a clear manifestation of overdrainage in adults, and thus should be included in the definition of overdrainage in adult patients.<br><br>*The vomiting worsens in the upright position.*	Disagree	
	Agree	
Radiological manifestations		
I find that "**slim or slit ventricles**" is a clear manifestation of overdrainage in adults, and thus should be included in the definition of overdrainage in adult patients.<br><br>*One could argue that slit ventricles is more specific for pediatric patients, or young adults with a pediatric onset, and thus not very descriptive in adult patients.*	Disagree	
	Agree	
I find that "**decreased ventricle size**" is a clear manifestation of overdrainage in adults, and thus should be included in the definition of overdrainage in adult patients.<br><br>*At the discussion meeting, slit ventricles and decreased ventricle size were in pooled in on group. I will allow myself to challenge you and ask you to rate 'a decreased ventricle size in comparison with previous imaging' alone.*	Disagree	
	Agree	
Measurable manifestation		
I find that "**low ICP**" is a clear manifestation of overdrainage in adults, and thus should be included in the definition of overdrainage in adult patients.<br><br>*What is normal age-related ICP?*<br>*Shall an invasive measurement be included in the definition, or optional for the physician?*<br>*Is low ICP in fact just a sign that the patient has underwent ICP measurement, and thus not a significant issue in terms of an OD-definition?*<br>*One could argue that the ICP-drop seen in a 'situp test' and thus not the absolute ICP value is more accurate.*	Disagree	
	Agree	
Manifestations related to shunt complications		

1. Four-Year Experience with the Routine use of the Programmable Hakim Valve in the Management of Children with Hydrocephalus
2. Effects of head elevation on intracranial hemodynamics in patients with ventriculoperitoneal shunts
3. Hydrocephalus: Overdrainage by Ventricular Shunts. A Review and Recommendations

Adult patients

I find that ”**risk of ventricular catheter obstruction due to collapse of the ventricular wall**” is a clear manifestation of overdrainage in adults, and thus should be included in the definition of overdrainage in adult patients.<br><br>*Based on the discussion meeting, ‘risk of shunt obstruction’ has been modified to the above mentioned.*<br><br>*A patient with OD might be in risk of (intermittent) ventricular catheter obstruction.*<br>*A patient with slit ventricles, a well regulated ICP, and an overdrainage protective device, is still in risk of ventricular catheter obstruction (due to collapse of the ventricular wall) but this does not equal that the patient suffers from OD.*<br>*One could argue whether this should be included in the definition of OD or if it is a manifestation that some patients with OD might experience.*<br>*One could further argue that ‘a risk of …’ is not a manifestation itself and thus should not be included.*	Disagree	
	Agree	
I find that ”**new onset of unilateral ventricular collapse on site of the implanted shunt +/- enlargement of the contralateral ventricle**” is a clear manifestation of overdrainage in adults, and thus should be included in the definition of overdrainage in adult patients.<br><br>*I will allow myself to challenge you, and ask you to rate the above mentioned, and see if you find that more or less correct than ‘risk of ventricular catheter obstruction due to collapse of the ventricular wall’.*	Disagree	
	Agree	
I find that ”**improperly shunt settings**” is a clear manifestation of overdrainage in adults, and thus should be included in the definition of overdrainage in adult patients.<br><br>*What is correct/proper shunt setting? No consensus could be reached at the meeting.*	Disagree	
	Agree	
I find that ”**an excessive siphoning effect**” is a clear manifestation of overdrainage in adults, and thus should be included in the definition of overdrainage in adult patients.<br><br>*Based on the discussion meetings, ‘siphoning effect’ has been modified to the above mentioned.*<br><br>*Siphoning is a physical mechanism which describes the increased CSF flow in an upright position.*	Disagree	
	Agree	
Syndromes		
I find that ”**slit ventricles syndrome**” is a clear manifestation of overdrainage in adults, and thus should be included in the definition of overdrainage in adult patients.<br><br>*One could argue that slit ventricle syndrome is more specific for pediatric patients, or young adults with a pediatric onset.*	Disagree	
	Agree	
I find that ”**low intracranial pressure syndrome**” is a clear manifestation of overdrainage in adults, and thus should be included in the definition of overdrainage in adult patients.<br><br>*In the paper suggesting ‘low intracranial pressure syndrome as a part of an OD-definition the term is mentioned in the following section and not further described. “We defined overdrainage and underdrainage as follows: Overdrainage complications: 1) Subdural hematomas or hygromas with a width of more than 4 mm or 2) Extreme reduction of ventricular size in presence of clinical symptoms (headache, vomiting, and nausea) more than 1 week after shunting or 3) Slit-ventricle syndrome (low intracranial pressure syndrome).”*<br><br>*A search in pubmed gave me 8 hits, 1 was not available, two was not in English, and two did not described the syndrome further. I’ve extracted the below mentioned descriptions of ‘low intracranial pressure syndrome’ from*<br><br>*- A low intracranial pressure (ICP) syndrome was suspected, because of the occurence of headache, nausea or vomiting in an erect position.*[1]<br><br>*- Low-ICP syndrome is characterized by headache, nausea, vomiting, lethargy, and even diplopia and paresis of upward gaze. It is usually associated with the upright position, and it is frequently relieved by lying down.*[2]<br><br>*- The low ICP syndrome, at times called negative pressure syndrome. It is characterized by headache, nausea, emesis, lethargy, and even diplopia and paresis of upward gaze with strabismus and vision impairment, usually associated with the upright position, and frequently relieved by lying down if such observations are routinely carried out.*[3]<br><br>*As none of the participants at the meeting were familiar with ‘low intracranial pressure syndrome’ one could argue that this in fact is too rare to be included in an OD definition.*	Disagree	
	Agree	

1. Four-Year Experience with the Routine use of the Programmable Hakim Valve in the Management of Children with Hydrocephalus
2. Effects of head elevation on intracranial hemodynamics in patients with ventriculoperitoneal shunts
3. Hydrocephalus: Overdrainage by Ventricular Shunts. A Review and Recommendations

References

1. Pedersen SH, Hannibal Prein, Tobias Ammar A, Grotenhuis A, Hamilton MG, Kehler U, Skovbo Hansen T, Rekate H, Thomale U, Juhler M (2023) How to define CSF overdrainage: a systematic literature review. Acta Neurochir. (Wien).
